# Clique-based data mining for related genes in a biomedical database

**DOI:** 10.1186/1471-2105-10-205

**Published:** 2009-07-01

**Authors:** Tsutomu Matsunaga, Chikara Yonemori, Etsuji Tomita, Masaaki Muramatsu

**Affiliations:** 1 Research and Development Headquarters, NTT DATA Corporation, Tokyo, 135-8671, Japan; 2 The Advanced Algorithms Research Laboratory, The University of Electro-Communications, Tokyo, 182-8585, Japan; 3 Research and Development Initiative, Chuo University, Tokyo, 112-8551, Japan; 4 Medical Research Institute, Tokyo Medical and Dental University, Tokyo, 101-0062, Japan; 5 Research Institute, HuBit Genomix Inc, Tokyo, 102-0092, Japan

## Abstract

**Background:**

Progress in the life sciences cannot be made without integrating biomedical knowledge on numerous genes in order to help formulate hypotheses on the genetic mechanisms behind various biological phenomena, including diseases. There is thus a strong need for a way to automatically and comprehensively search from biomedical databases for related genes, such as genes in the same families and genes encoding components of the same pathways. Here we address the extraction of related genes by searching for densely-connected subgraphs, which are modeled as cliques, in a biomedical relational graph.

**Results:**

We constructed a graph whose nodes were gene or disease pages, and edges were the hyperlink connections between those pages in the Online Mendelian Inheritance in Man (OMIM) database. We obtained over 20,000 sets of related genes (called 'gene modules') by enumerating cliques computationally. The modules included genes in the same family, genes for proteins that form a complex, and genes for components of the same signaling pathway. The results of experiments using 'metabolic syndrome'-related gene modules show that the gene modules can be used to get a coherent holistic picture helpful for interpreting relations among genes.

**Conclusion:**

We presented a data mining approach extracting related genes by enumerating cliques. The extracted gene sets provide a holistic picture useful for comprehending complex disease mechanisms.

## Background

Progress in the life sciences has recently been made by integrating biomedical knowledge on numerous genes and formulating hypotheses on the genetic mechanisms underlying various vital phenomena [1,2]. A large variety of genetic and biomedical knowledge on genes has been compiled into databases [3], and is available in electronic forms such as the Online Mendelian Inheritance in Man (OMIM) database [4]. Researchers and physicians formulating hypotheses often need to identify groups of functionally related genes, such as gene families and gene pathways, and this is usually done by simply reading a large number of documents related to the phenomenon of interest [5]. Since such an approach will inevitably result in some relevant literature being overlooked, researchers and physicians need a way that will help them search for related gene sets automatically and comprehensively [6].

Graph-based approaches [7-9] have recently emerged as a method for data mining. A biomedical relational graph is formed by nodes that represent biological entities (e.g. genes/proteins) and edges that represent the associations of those entities. For instance, protein-protein interactions are modeled by a graph, where nodes are proteins and two nodes are connected by an edge if the corresponding proteins physically bind. Protein functions are predicted using connections in a graph [10] based on the assumption that proteins which lie close to one another are more likely to have similar functions or constitute protein complexes. For extracting coherent groups of genes as modules, a module-assisted approach [11,12] has been introduced. Prior studies include attempts to extract modules from protein-protein interactions [13,14], co-expression in microarray data [15,16], and gene symbol co-occurrence in Medline article abstracts [17]. Computational tools for visualizing modules (sets of nodes) in a given graph [18] have been developed.

Here we have constructed a biomedical relational graph whose nodes are pages of genes or diseases and whose edges are hyperlink connections between pages by using over 10,000 entities in the OMIM database [4]. The OMIM database, which is a biomedical database of human genes and genetic disorders, contains a great number of relationships between genes and diseases. This work was based on the assumption that the structures of hyperlink connections correspond to the structural features of biological systems. Clique-based data mining has been applied to a relational graph based on the assumption that relevant relationships are reflected in completely interconnected subgraphs (cliques) or nearly completely interconnected subgraphs (pseudo-cliques). We address the extraction of related genes (called 'gene modules' in this paper) by searching for densely connected subgraphs in a biomedical relational graph. Sets of related genes are detected by enumerating densely-connected subgraphs modeled as cliques [19-21] or pseudo-cliques [22,23]. Using this method, we extracted over 20,000 gene modules. To the best of our knowledge, this is the first study to show that sets of related genes can be comprehensively extracted from a biomedical database and that these related genes can be utilized to gain insight into the mechanisms of complex diseases.

## Methods

### Materials

The experimental materials were taken from the Online Mendelian Inheritance in Man (OMIM) database, a well-known catalog in which human genes and genetic disorders are assigned descriptive code numbers [24]. Using these numbers, they are connected by hyperlinks according to their associations, such as physical proximity, similarity of nomencalture or structure, or functional association.

KEGG pathway data that contains sets of genes were obtained (November 2003) from a database produced by the Cancer Genome Anatomy Project [25].

### Clique enumeration

An undirected graph *G *consists of a set *V *of nodes and a set *E *of non-weighted edges connecting pairs of nodes. The number of edges connected to node *v *∈ *V *is referred to as the degree of node *v *in *G*. The subgraph of *G *is induced by the subset *V' *of *V*. A subgraph in which every pair of nodes is connected by an edge (i.e., a complete subgraph) is called a clique, and the size of which is the number of nodes in it. A clique is called maximal if it is included in no other clique. Pseudo-cliques are the subgraphs obtained by relaxing the connectivities, and the connectivity is measured by the edge density *ρ*. The edge density of a pseudo-clique *S *is the ratio of the number of edges in *S *to the number of edges in a clique that has the same number of nodes that *S *does. It is calculated as follows:



where |*E*(*S*)| is the number of edges in *S *and |*S*| is the number of nodes in *S*. By setting *ρ*(*S*) to a threshold value *θ *(0 ≤ *θ *≤ 1) pseudo-cliques whose edge density is not less than *θ *can be enumerated [26]. As a *θ *decreases from 1 to 0, we get pseudo-cliques whose connectivity is more and more relaxed.

In the work presented in this paper we obtained gene modules (sets of biologically related genes) by enumerating pseudo-cliques in a graph whose node were genes and genetic disorders and whose connecting edges were biomedical relations.

### Correspondence analysis

Correspondence analysis [27] is a method to analyze relations between categorical variables, called cases and items. This analysis yields an arrangement in which similar cases and items are closely placed. By introducing a data matrix whose rows and columns are variables (cases and items) having values of 0 or 1 at components depending on the absence or presence of the relations between the variables, rows (items) and columns (cases) are arranged by sorting the scores calculated using the second largest eigenvalue and the corresponding eigenvector.

In the work presented in this paper items and cases are assigned respectively to modules and genes/diseases contained.

## Results and discussion

### An example: enumerating cliques

We will present an example of enumerating cliques from a part of an undirected graph used in experiments described later in this paper. This enables us to see clique enumeration from a biomedical relational graph and to investigate the validity in an accessible fashion.

**Figure 1:**
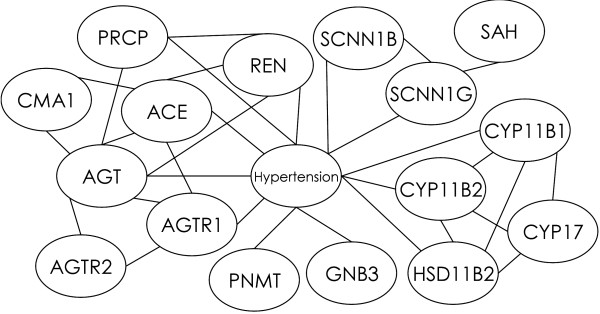
**Example of a biomedical relational graph**. Hypertension and hypertension-related genes are represented by nodes, and the associations between them are represented by edges.

Figure 1 shows a biomedical relational graph in which hypertension and 16 hypertension-related genes are represented by nodes and in which the biomedical associations between pairs of genes and between genes and hypertention are represented by edges. The graph has 17 nodes and 31 edges. It is understandable that a computational device is required in order to grasp the structure of relationship among genes and diseases as the scale becomes large.

The node sets for all the maximal cliques obtained from the graph in Figure 1 are listed in Table 1. It is expected that there are many cliques that contain the hypertension node. The extraction of cliques whose nodes include hypertension and the AGT, ACE, AGTR1, and REN genes, is in agreement with the medical knowledge. The renin-angiotensin system in which the REN, AGT, ACE, and AGTR1 genes interact with each other [28] is the well known pressor mechanism that acts in concert with the CYP11B1 and CYP11B2 genes related to aldosterone secretion. The extraction of cliques whose nodes include the CYP17 and HSD11B2 genes in addition to the CYP11B1 and CYP11B2 genes is consistent with the reported interaction of their enzymes with aldosterone synthesis [29]. Related genes that regulate each other can thus be extracted by enumerating cliques in biomedical relational graphs.

**Table 1:** All maximal cliques obtained from the graph in Figure 1

Maximal clique	Size
{CMA1, AGT, ACE}	3
{Hypertension, AGT, ACE, AGTR1}	4
{Hypertension, AGT, ACE, REN}	4
{Hypertension, AGT, PRCP, REN}	4
{Hypertension, CYP11B2, CYP11B1, HSD11B2}	4
{Hypertension, GNB3}	2
{Hypertension, PNMT}	2
{Hypertension, SCNN1B, SCNN1G}	3
{SAH, SCNN1G}	2
{CYP17, CYP11B2, CYP11B1, HSD11B2}	4
{AGTR2, AGT, AGTR1}	3

### Biomedical relational graph by OMIM hyperlinks

We constructed an undirected graph whose nodes were gene or disease pages in the OMIM database and whose edges were hyperlink connections between those pages. As shown by the data listed in Table 2, the graph had 13,722 nodes and 35,749 edges (as of December 2001). Each hyperlink connection was counted only once. We limited this study to 6,010 genes both in the OMIM database and in Swiss-Prot [30] (November 2002), excluding genes with no Medline citations or no gene locus descriptions or no hyperlinks to other OMIM pages. The characteristic path length is defined as the number of edges in the shortest path between two nodes, averaged over all pairs of nodes. The clustering coefficient measures the average degree of node coherence connected by the edges. When a node *V *has *k*_*v *_connected nodes, the clustering coefficient is the ratio of the actual number of edges to the possible *k*_*v*_(*k*_*v*_-1)/2 edges. The characteristic path length and clustering coefficient [31] were respectively 4.99 and 0.27 (Table 2). These values indicate that the graph contained clusters of densely-connected nodes and that there were hub-like nodes connecting the clusters.

**Table 2:** Structural properties of the graph used in the experiment

Number of nodes	13,722
Number of edges	35,749
Average degree	5.23
Characteristic path length	4.99
Clustering coefficient	0.27

### Extraction of gene modules by searching cliques

In the graph were 20,486 maximal cliques and the largest maximal clique contained 12 nodes. The 20 most frequent genes in the maximal cliques are listed in Table 3 along with their degrees and the number of times they were found in cliques of various sizes. The NFKB1 gene (the 3rd most frequent gene, or #3) was also in five cliques of size 10, the BRCA1 gene (#11) in one clique of size 12, and the TAF1 gene (#19) in one clique of size 8. The genes found most frequently in the maximal cliques (such as TNF, TP53, and NFKB1) are typical genes that play a central role and are prevalent research subjects. The clique distribution of the TAF1 gene (#19) is relatively shifted towards larger sizes, suggesting it forms complexes with some gene products since the gene function is a transcription factor.

**Table 3:** The 20 most frequent genes in the maximal cliques.

				Size						
Rank	Gene	Degree	Total	2	3	4	5	6	7	8–12
1	TNF	176	244	19	89	64	44	20	8	0
2	TP53	182	220	31	118	52	17	1	1	0
3	NFKB1	153	217	15	57	53	53	25	9	5
4	TGFB1	123	136	35	48	38	8	7	0	0
5	IFNG	128	115	19	34	33	21	7	1	0
6	TNFRSF1A	70	115	3	20	39	37	12	4	0
7	RB1	103	112	17	48	32	11	4	0	0
8	HD	102	105	20	57	19	3	6	0	0
9	MYC	111	104	26	62	9	6	1	0	0
10	HRAS	103	103	26	50	26	0	1	0	0
11	BRCA1	98	97	20	37	29	8	2	0	1
12	APC	84	94	13	53	24	4	0	0	0
13	MAPK8	67	91	3	25	41	16	5	1	0
14	EGF	97	85	32	38	12	3	0	0	0
15	TNFRSF6	66	81	7	24	28	22	0	0	0
16	MEN1	69	74	21	34	17	2	0	0	0
17	GH1	73	73	8	25	29	9	2	0	0
18	NF1	76	73	19	39	13	1	1	0	0
19	TAF1	46	71	4	9	6	27	13	11	1
20	IL1B	72	70	13	32	18	7	0	0	0

Also listed for each gene is its degree and the number of times it was found in cliques of various sizes.

When pseudo-cliques were extracted by the relaxing edge connecting condition, the maximum sizes increased as *θ *decreased (Table 4). Preliminary experimental results showed that gene modules should consist of possible related genes for the analysis. By relaxing connectivities (see Methods), pseudo-cliques were introduced for gene modules to collect the possible related genes. The maximum pseudo-clique size reached 14 when *θ *= 0.88 and did not increase further even when *θ *was decreased to 0.7 (data not shown).

**Table 4:** Number of cliques corresponding to *θ *values

Size\*θ*	1.0 (complete)	0.96	0.92	0.88
14	-	-	-	1
13	-	-	5	27
12	2	2	2	26
11	1	11	6	31
10	6	2	1	75
9	4	11	17	394
8	11	66	447	452
7	49	16	53	347
6	188	188	1597	227
5	712	712	168	6352
4	2188	2188	2188	385
3	6330	6330	6330	6330
2	10995	10995	10995	10995

Total	20486	20521	21809	25642

For this reason, 25,642 pseudo-cliques (*θ *= 0.88) were taken as gene modules in the following analysis. The sensitivity and specificity of the current method in extracting biologically relevant genes using cliques can not be readily assessed. One would assume that cliques with larger size are likely to be more biologically relevant. To account for this, we estimated whether a cutoff value on the clique size can be set. We examined the maximum size of cliques in randomized graphs having common node degrees to the OMIM hyperlink graph. In the experiments of enumerating pseudo-cliques (*θ *= 0.88) in the randomized graphs which were generated using an edge-swapping approach [32], the maximum size four was observed (data not shown). This result suggests that clique size four may be considered as a background clique size, and that clique size of more than four may be biologically relevant.

While we employed the clique enumeration method to extract sets of related genes, application of the method by the edge-betweenness clustering [11,12], which introduces the concept of graph modularity called community, may also be considered. Basically the community-based approach is conducted with graph partitioning to separate connected nodes into groups of nodes that have a high density of edges within them, with a lower density of edges between groups [13]. In contrast, the clique-based approach allows for any node to belong to more than one group of nodes. It would be interesting to compare the performance of these different methods and investigate how to use the methods properly.

### Biological evaluation of extracted gene modules

To evaluate how well the gene modules correspond to known gene pathways, we compared the sets of genes in the modules with those in the KEGG pathway database [33]. The 66 KEGG pathways that contain more than five genes and the 25,642 gene modules (*θ *= 0.88) were compared by calculating values of the Jaccard's coefficient *r*_*J *_(0 ≤ *r *_*J *_≤ 1). The Jaccard's coefficient is often used as a criterion when evaluating the similarity of two sets, and is the ratio of the size of the intersection of the sets to the size of the union of the sets. Its value thus approaches 1 as the extent of coincidence increases. The 15 most relevant KEGG pathways and the corresponding gene module sizes are listed in Table 5, where the numbers in the square brackets are numbers of genes in the KEGG pathway. For instance, in the top rank of KEGG pathway 'Blood Group Glycolipid Biosynthesis,' the number of genes contained was six. The corresponding gene module by a pseudo-clique included four genes. As for the other KEGG pathways, none of the gene module sizes equaled the number of genes in the KEGG pathway. This implies that a pseudo-clique can partially extract genes included in biological pathways.

**Table 5:** The 15 KEGG pathways most relevant to the extracted gene modules

Rank	KEGG pathway [number of genes]	Gene module size	*r*_*J*_(intersection/union)
1	hsa00601 Blood Group Glycolipid Biosynthesis – Lact Series [6]	4	0.667 (4/6)
2	hsa00920 Sulfur Metabolism [7]	4	0.571 (4/7)
3	hsa00532 Chondroitin/Heparan Sulfate Biosynthesis [8]	6	0.556 (5/9)
4	hsa00140 C21-Steroid Hormone Metabolism [11]	7	0.500 (6/12)
5	hsa00040 Pentose and Glucuronate Interconversions [10]	5	0.500 (5/10)
6	hsa00400 Phenylalanine, Tyrosine and Tryptophan Biosynthesis [8]	3	0.375 (3/8)
7	hsa00511 Glycoprotein Degradation [8]	3	0.375 (3/8)
8	hsa03050 Proteasome [14]	5	0.357 (5/14)
9	hsa03020 RNA Polymerase [15]	5	0.333 (5/15)
10	hsa00530 Aminosugars Metabolism [9]	3	0.333 (3/9)
11	hsa00580 Phospholipid Degradation [9]	3	0.333 (3/9)
12	hsa00062 Fatty Acid Biosynthesis (path 2) [6]	2	0.333 (2/6)
13	hsa00602 Blood Group Glycolipid Biosynthesis – Neolact Series [6]	2	0.333 (2/6)
14	hsa00271 Methionine Metabolism [10]	3	0.300 (3/10)
15	hsa00790 Folate Biosynthesis [10]	3	0.300 (3/10)

In order to evaluate how well the gene modules accord with interactions between genes, we compared the sets of genes in the modules with gene pairs in the protein-protein interaction data [34]. The 650 interactions for the above-mentioned 6,010 genes were obtained from the protein-protein interaction data. Among the 650 gene pairs 145 pairs were captured in the gene modules, which correspond to 22.3% of the total interactions. The gene pairs in the protein-protein interactions which were repeatedly captured by gene modules are listed in Table 6. The gene pair in the first row is involved in genes of biological pathways related to MAPK signaling and apoptosis. The second pair is involved in TGF-*β *signaling. The third is in regulation of food intake and energy monitoring. Repeated captures suggest that the pairs have various functions in biological setting.

**Table 6:** The captured gene pairs in the protein-protein interaction database

Rank	Number of times	Gene pair		
1	70	AKT1	-	CHUK
2	38	ACVRL1	-	TGFB1
3	24	AGRP	-	MC3R
4	21	BAK1	-	BAX
5	17	CASP10	-	FADD
6	15	BAX	-	BCL2L1
6	15	BMPR1A	-	BMPR1B
8	14	ACVRL1	-	TGFBR2
8	14	CASP10	-	CFLAR
8	14	CASP8	-	FADD
11	13	APC	-	AXIN2
12	12	BAK1	-	BCL2L1
13	11	A2M	-	APOE
13	11	ABL1	-	BCR
13	11	APAF1	-	CASP9
13	11	ARNTL	-	CLOCK
13	11	CASP8	-	CASP10

Table 7 shows gene sets included in typical large gene modules. The gene module in the first row is constituted by a family of chemokine genes, and the gene module in the second comprises NF-*κ*B family genes (including RelA and RelB) and genes that form complexes with them (I*κ*B). The gene module in the third row is made up of 'DNA repair'-related genes. The BRCA1-associated proteins; the BLM, MSH6, MSH2, and MLH1 proteins; and subunits of the RFC complex are involved in DNA repair [35]. The genes in the module in the fourth row are related to general transcription factor (GTF) protein complexes. The gene module in the bottom row is associated with the signal transduction pathway of the inflammatory response [36]. TNF receptor-associated factor 2 (TRAF2) is a protein that interacts with TNF receptors and is required for signal transduction. The MAP kinase kinase kinase 14 (MAP3K14) gene in this module encodes a protein that simulates NF-*κ*B activity by binding to the TRAF2 gene product. The gene modules thus comprise various types of related genes including gene families, complexes, and pathways. As illustrated above, the current results have biological coherence for analyzing relations among genes.

**Table 7:** Typical large gene modules computationally extracted as pseudo-cliques

Gene module	Attribute
{PPBP, SCYB6, GRO2, GRO3, IL8, SCYB10, IFNG, GRO1, PF4, SCYB5, MIG, SCYB11}	Family
{NFKBIA, NFKB1, NFKB2, RELA, REL, CHUK, MAP3K7, IKBKB, NFKBIB, MAP3K14, RELB}	Family & Complex
{RFC4, RFC1, BRCA1, MSH2, MLH1, APC, RFC2, MSH6, MRE11A, BLM}	Complex
{POLR2A, GTF2E1, GTF2B, GTF2F1, GTF2H1, TAF1, TAF10, GTF2A2, GTF2A1}	Complex
{TNFRSF5, NFKB1, TNF, TNFRSF1A, TNFRSF1B, CHUK, TRAF2, MAP3K14}	Pathway

### Analysis of gene relationships using 'metabolic syndrome'-related gene modules

For applying gene modules to disease mechanism analysis, we assembled gene modules associated with the metabolic syndrome [37] as an example of a typical multifactorial disease. The metabolic syndrome is a heterogeneous disease characterized by the onset and progession of four common disorders: obesity, diabetes, hyperlipidemia, and hypertension. The genes associated with these disorders might interact with each other and lead to arteriosclerotic diseases such as myocardial infarction or ischemic stroke. Public attention has been focused on its prevention [38]. We examined the congruence with current medical knowledge.

Gene modules associated with diabetes, hyperlipidemia, hypertension, and obesity are obtained by their containing the disease nodes, which are non-insulin-dependent diabetes mellitus (MIM 125853), familial combined hyperlipidemia (144250), essential hypertension (145500), and obesity (601665). Out of 25,642 modules, 110, 16, 34, and 28 modules are obtained, respectively. There were no overlaps among the modules. Then a total of 188 modules and 124 genes contained were identified.

The 10 most frequent genes in the 188 modules are listed in Table 8 along with the numbers of times they were found in the modules (i.e., cliques) of various sizes. As shown in the table, INS gene and LEP gene are the top and the 2nd, respectively. The modules of size 6 including INS gene or LEP gene were {Obesity, LEP, MC4R, POMC, AGRP, LEPR}, {Obesity, LEP, MC4R, POMC, AGRP, PCSK1}, {Diabetes, LEP, IGF1, IRS1, INS, IRS2}. Each module contains biologically plausible genes related to obesity or diabetes.

**Table 8:** The 10 most frequent genes in the 188 extracted modules associated with the metabolic syndrome

			Size					
Rank	Gene	Total	2	3	4	5	6	7
1	INS	29	2	6	2	18	1	0
2	LEP	27	2	6	4	12	3	0
3	POMC	16	1	0	3	10	2	0
4	PCSK1	13	0	1	2	9	1	0
5	IRS2	12	0	0	0	11	1	0
5	IGF1	12	0	1	0	10	1	0
5	INSR	12	3	4	1	4	0	0
8	IRS1	11	0	0	1	9	1	0
9	MC4R	10	0	0	1	7	2	0
10	FGF1	9	0	0	1	4	2	2

**Figure 2:**
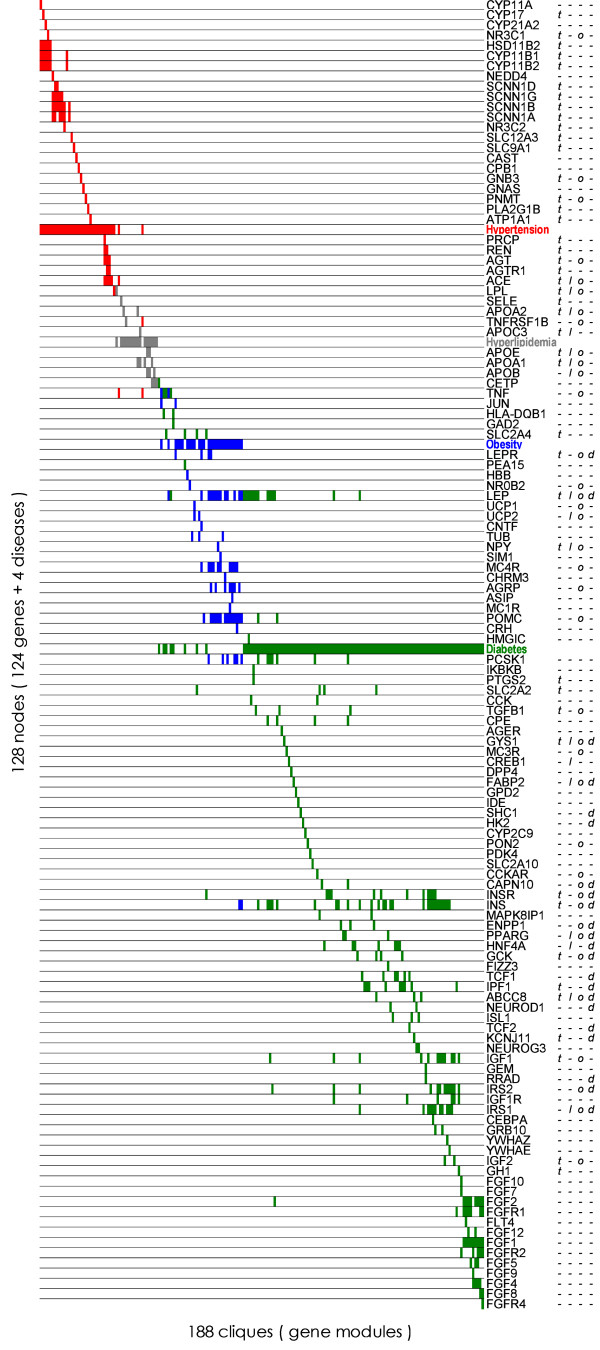
**188 modules associated with obesity, diabetes, hyperlipidemia, and hypertension**. The vertical bars indicate the genes/diseases in the modules, the columns represent modules, and the rows represent the genes/diseases. The rows and columns are sorted in the ascending order by the score calculated by correspondence analysis (see Methods). Red, grey, blue, and green bars respectively indicate cliques that contain the hypertension, hyperlipidemia, obesity, and diabetes nodes. The letters 't,' 'l,' 'o,' and 'd' on the right show that in the literature the genes are related to hypertension, hyperlipidemia, obesity, and diabetes (see text for the literature references).

We combined the 188 modules and 124 genes using correspondence analysis (Fig. 2). Each bar in Figure 2 corresponds to a gene node or disease node in the cliques, and the names of the genes or diseases are shown on the right. Each bar is colored according to the disease nodes included in the corresponding module: red for hypertension, gray for hyperlipidemia, blue for obesity, and green for diabetes. The letters of 't,' 'l,' 'o,' and 'd' to the right of the figure respectively indicate that in the literature the genes in the corresponding rows are related to hypertension [39], hypertrigricedemia [40], obesity [41], and diabetes [42].

Hyperlipidemia-related genes are replaced by genes associated with hypertrigricedemia (hyperlipidemia together with hypercholesterolemia). As shown in Figure 2, the gene modules associated with the four diseases occupy their own regions from the top-left to the bottom-right. As for the disease nodes shown to be belt-like in the figure, the nodes of hypertension, hyperlipidemia, obesity, diabetes are placed from the top to the middle. There is a diabetes node at the middle and the letter 'd' indicating diabetes-related genes are mostly clustered in the lower half. This implies that gene modules properly reflect the biological functions of the corresponding genes and that the modules can provide a holistic view of a complex disease. Correspondence analysis has been successfully applied to combine gene modules for interpreting relationships among genes with their gradation on relation to diseases.

Genes related to the above-mentioned aldosterone metabolism (CYP17, HSD11B2, CYP11B1, CYP11B2) are at the top of the figure. Genes related to the renin-angiotensin system (REN, AGT, AGTR1, ACE) are around the hypertension node indicated by the cluster of 't' marks, and apolipoprotein genes (APOA2, APOC3, APOE, APOA1, APOB) are gathered around the hyperlipidemia node close to the 'renin-angiotensin system'-related genes. It is worth noting that 'food intake regulation'-related genes such as LEP, NPY, MC4R, AGRP and POMC genes are grouped comprehensively (i.e., without overlooking relevant genes). Insulin resistance is defined as a status in which the action of insulin is insufficient and proper energy conversion is impaired. The TNF gene related to insulin resistance [43], which is a shared foundation of the four disorders, was in modules associated with hypertension, obesity, and diabetes, indicating that it has a variety of effects on the development of metabolic syndrome. It would be intriguing to find the CRH gene, which has a biological function in stress responses based on the hypothalamic-pituitary-adrenal axis, in the vicinity of the 'food intake regulation'-related gene region. FGF (fibroblast growth factor)-family genes appear at the bottom of the figure, indicating their relevance to diabetes. Although FGF-genes are associated with cancers, inflammation has been investigated in both cancer and metabolic syndrome. Such findings in this chart suggest innovative avenues of research. Obtaining the comprehensive list of related genes makes it possible to combine the gene modules and grasp relations among genes quantitatively, facilitating hypothesis formulation from a holistic viewpoint. In principle, the biological functions of desired genes as well as disease-related genes could be grasped by combining modules that contain those genes.

## Conclusion

We have shown that related genes can be extracted comprehensively by enumerating pseudo-cliques in biomedical relational graph. Over 20,000 gene modules that include genes in the same family, genes encoding proteins in the same complexes, and genes encoding components of the same signaling pathway were extracted automatically. Furthermore these gene modules were utilized for visualizing relations between genes and diseases. Extraction of related genes (referred to as gene modules in this paper) would be more important since most biomedical tasks are performed not by individual genes, but by sets of functionally associated genes. The method using pseudo-cliques can generally extract related gene sets under a single computational operation although the application is restricted to genes connected by their relations in the manner of the OMIM hyperlink connections. For clarifying complex disease mechanisms, obtaining relationships among genes associated with the diseases should be crucial. Comprehensive extraction of related-gene sets by clique-based data mining may provide us with a systematic methodology for gaining insight into the genetic mechanisms underlying various biological phenomena including diseases.

## Authors' contributions

TM and ET conceived and designed the experiments. TM and CY performed the experiments. TM and MM analyzed the data. All authors have read and approved the final manuscript.
